# The Specificities of Thrombotic Thrombocytopenic Purpura at Extreme Ages: A Narrative Review

**DOI:** 10.3390/jcm12093068

**Published:** 2023-04-23

**Authors:** Adrien Joseph, Bérangère S. Joly, Adrien Picod, Agnès Veyradier, Paul Coppo

**Affiliations:** 1 Medical Intensive Care Unit, Saint-Louis Hospital, Public Assistance Hospitals of Paris, 75010 Paris, France; adrien.joseph@aphp.fr (A.J.); adrien.picod@aphp.fr (A.P.); 2 French Reference Center for Thrombotic Microangiopathies, 75012 Paris, France; berangere.joly@aphp.fr (B.S.J.); agnes.veyradier@aphp.fr (A.V.); 3 Hematology Biology Department, Lariboisière Hospital, Public Assistance Hospitals of Paris, 75006 Paris, France; 4 EA-3518, Clinical Research in Hematology, Immunology and Transplantation, Institut de Recherche Saint-Louis, Université de Paris, 75571 Paris, France; 5 Hematology Department, Saint-Antoine hospital, Public Assistance Hospitals of Paris, 75571 Paris, France

**Keywords:** thrombotic microangiopathy, thrombotic thrombocytopenic purpura, ADAMTS13 protein, diagnosis, prognosis, child, aging, plasma exchange, caplacizumab, von Willebrand factor

## Abstract

Thrombotic thrombocytopenic purpura (TTP) is a rare and life-threatening thrombotic microangiopathy (TMA) related to a severe ADAMTS13 deficiency, the specific von Willebrand factor (VWF)-cleaving protease. This deficiency is often immune-mediated (iTTP) and related to the presence of anti-ADAMTS13 autoantibodies that enhance its clearance or inhibit its VWF processing activity. iTTP management may be challenging at extreme ages of life. International cohorts of people with TTP report delayed diagnoses and misdiagnoses in children and elderly people. Child-onset iTTP shares many features with adult-onset iTTP: a female predominance, an idiopathic presentation, and the presence of neurological disorders and therapeutic strategies. Long-term follow-ups and a transition from childhood to adulthood are crucial to preventing iTTP relapses, in order to identify the occurrence of other autoimmune disorders and psychosocial sequelae. In contrast, older iTTP patients have an atypical clinical presentation, with delirium, an atypical neurological presentation, and severe renal and cardiac damages. They also have a poorer response to treatment and prognosis. Long-term sequelae are highly prevalent in older patients. Prediction scores for iTTP diagnoses are not used for children and have a lower sensitivity and specificity in patients over 60 years old. ADAMTS13 remains the unique biological marker that is able to definitely confirm or rule out the diagnosis of iTTP and predict relapses during follow-ups.

## 1. Introduction

Thrombotic thrombocytopenic purpura (TTP) is a thrombotic microangiopathy caused by a severe functional deficiency in a disintegrin and metalloprotease with thrombospondin type I repeats-13 (ADAMTS13), the specific von Willebrand factor (VWF)-cleaving protease [1]. TTP is associated with microangiopathic hemolytic anemia, severe thrombocytopenia, and end-organ ischemia, which is linked to the spontaneous formation of microvascular VWFs and platelet-rich thrombi, particularly in the central nervous system [2].

In its most frequent form, immune-mediated TTP (iTTP) is caused by autoantibodies, mainly IgG, which are directed against ADAMTS13, where they inhibit its function or enhance its clearance. The anti-ADAMTS13 autoimmune response is polyclonal. Similar to other autoimmune diseases [3], iTTP predominantly affects women of reproductive age (30–40 years). In its presentation, iTTP is idiopathic in 60% of cases and is associated with another preexisting or concomitant clinical condition in 40% of cases [4]. In its non-idiopathic forms, infections, systemic autoimmune diseases, cancer, transplantation, antiplatelet drugs, immunosuppressive agents, HIV, and pregnancy are the most commonly listed triggers for iTTP [4].

French and PLASMIC scores, both of which are based on platelet counts and serum creatinine levels at diagnosis, have been developed for the early identification of adult patients with severe ADAMTS13 activity, in order to guide the clinical and therapeutic decisions when ADAMTS13 testing is not available in an emergency [5,6]. The measurement of ADAMTS13 activity is crucial to confirming an iTTP diagnosis (activity <10 IU/dL) [1,7]. Additional investigations into the presence of anti-ADAMTS13 autoantibodies (mainly ELISAs to detect anti-ADAMTS13 IgG) are required to document the auto-immune mechanism of ADAMTS13 deficiency [2,7,8].

**Figure 1:**
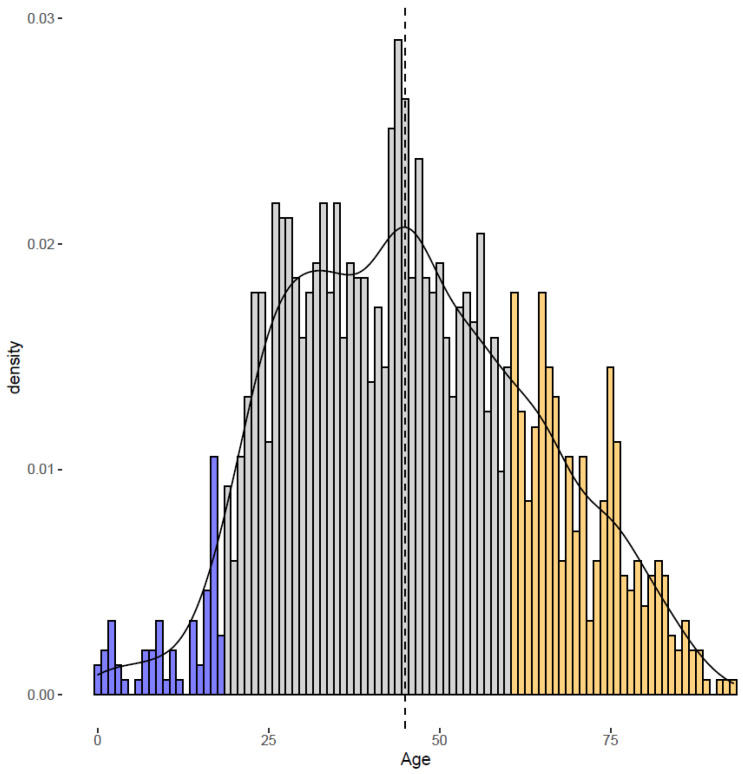
Distribution of immune-mediated thrombotic thrombocytopenic purpura cases according to age of onset.

Due to its rarity (a prevalence of around 5–13 cases/1,000,000) [4,9,10], the initial description of iTTP was focused on prototypical patients [11]. As larger cohorts were reported, it appeared clear that younger and older patients represented an appreciable share of iTTP patients. In the French National Registry for TTP, 4% of cases occurred in individuals below 18 years, and 17% to 23% of cases in individuals above 60 years (Figure 1). Moreover, differences in the phenotypes and outcomes of such patients were described.

The proportions of adulthood- (73%, grey), old-age- (23%, orange), and childhood (4%, blue)-onset iTTP that are presented in this figure have been extracted from the data of the French Registry for TTP (inclusion period 2000–2020, 1514 patients). The median age is represented as a dashed line.

In this review, we will describe the particular characteristics of iTTP occurring at extreme ages, i.e., below 18 years and above 60 years of age. We will also describe the implications in terms of their outcomes and the recommendations for providing optimal care to these patients and preventing delayed management.

## 2. Immune-Mediated Thrombotic Thrombocytopenic Purpura in Children

In 1924, Eli Moschcowitz described the first case of iTTP in a 16-year-old girl who had suddenly developed weakness, pain, pallor, fever, and petechiae (no platelet count available). A few days later, she developed neurological disorders and died. The autopsy revealed the presence of disseminated hyaline thrombi in the microcirculation of her heart, kidney, spleen, and liver [12]. Based on the scope of the scientific literature and the national registries for TTP since 2001, ~150 different cases of child-onset iTTP with a documented severe functional deficiency of ADAMTS13 (activity < 10 IU/dL per definition) and the presence of anti-ADAMTS13 autoantibodies have been reported [13,14,15,16,17,18,19,20,21,22,23,24,25]. The prevalence of child-onset iTTP is ~1 case per million children and its diagnosis remains challenging [13,25].

### 2.1. Clinical Presentation and Diagnosis

**Figure 2:**
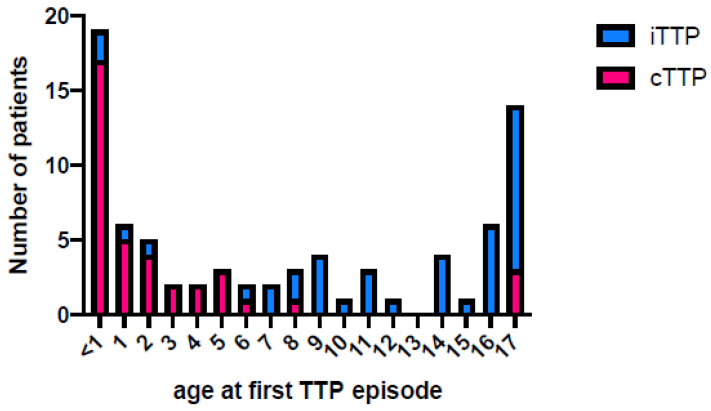
Proportion of child-onset congenital (cTTP) and immune thrombotic thrombocytopenic purpura (iTTP) according to age at first TTP episode.

Child-onset iTTP is rare and life-threatening. Besides an early onset in the neonatal period of the congenital form of TTP (~1/3 of child-onset TTP cases), iTTP may occur in ~2/3 of child-onset TTP cases, with a frequency that is two times greater for older compared to younger children [2,13,21,25] (Figure 2). Physicians have to rule out the possibility of congenital TTP before starting treatment for iTTP with immunosuppressive drugs or anti-VWF agents.

The proportions presented in this figure have been extracted from the data of the French Registry for TTP (inclusion period 2000–2020).

In children, the median age of the first iTTP episode is ~12 years and the sex ratio is ~2–2.5 F/1 M [13,14,15,16,17,18,19,20,21,22,23,24,25]. The clinical presentation of iTTP is mainly idiopathic (~56% with a median age of 15 years), but other clinical contexts such as infection, systemic autoimmune disease (mainly systemic lupus erythematosus), neoplasia, or organ transplantation are sometimes associated with its inaugural episode (~44% with a median age of 8 years) [13,21]. iTTP remains rare before 6 years.

In child-onset iTTP, fever and neurological symptoms (such as headache, confusion, coma, seizures, strokes, or transient focal defects) are frequent (~40% and 40–55%, respectively), while renal or cardiac injury are less common (~40% and ~6–7%, respectively) [13,14,15,16,17,18,19,20,21,22,23,24,25].

The most important laboratory features are severe consumption thrombocytopenia (a platelet count typically of <30 × 10^9^/L) and microangiopathic hemolytic anemia (hemoglobin levels usually of 6–7 g/dL), with the presence of schistocytes on the peripheral blood smear. The first episode of iTTP may be sudden and severe. By definition [7], all iTTP patients have an ADAMTS13 activity of less than 10 IU/dL and positive anti-ADAMTS13 autoantibodies at diagnosis [13,14,15,16,17,18,19,20,21,22,23,24,25].

In 20–25% of children, iTTP can be misdiagnosed as autoimmune cytopenia (idiopathic thrombocytopenic purpura, Evans syndrome) or another thrombotic microangiopathy (TMA) syndrome, such as shigatoxin-mediated hemolytic uremic syndrome (HUS) or atypical HUS, due to the dysregulation of the complement alternative pathway or a malignant hemopathy [13]. An iTTP diagnosis should be suspected when microangiopathic hemolytic anemia and consumption thrombocytopenia are associated with organ failure or a previous diagnosis of autoimmune cytopenia is not responding to specific treatments.

An ADAMTS13 activity measurement is the unique biological marker that is able to differentiate iTTP from other TMA syndromes or immune cytopenias [2,7]. In adult patients, both French and PLASMIC scores facilitate the rapid recognition of a severe ADAMTS13 deficiency and guide the clinical decisions when ADAMTS13 testing is not available [5,6]. The performances of these scores with age-related variables should be evaluated in children to improve iTTP diagnoses.

### 2.2. Treatment

The treatment of the acute phase of iTTP is an emergency because major stroke and organ failure can subsequently occur. The therapeutic targets used in child-onset iTTP are ADAMTS13, anti-ADAMTS13 IgG, and VWF [26].

A therapeutic plasma exchange (TPE) or plasma infusion, allowing for an exogenous supply of ADAMTS13 deficiency and the saturation of anti-ADAMTS13 autoantibodies, is the first-line treatment for acute iTTP in children, as soon as an iTTP diagnosis is made or even suspected [27].

Corticosteroids are usually used as an adjunctive treatment to curative first-line plasmatherapy. When iTTP is confirmed, an immunomodulation with rituximab (a chimeric anti-CD20 monoclonal antibody) may be considered together with TPE and corticosteroids to decrease the autoimmune response and normalize the ADAMTS13 levels [27]. Rituximab is typically effective after 2 weeks following the first infusion; therefore, it does not prevent early death [2].

Caplacizumab, a nanobody directed against the A1 domain of VWF, immediately inhibits the interactions between platelet GPIb and VWF and prevents the formation of microvascular thrombosis in the microcirculation. Caplacizumab has shown safety and efficacy in adult-onset iTTP [20,28]. Immunosuppressive therapies are still required to control the underlying disease process [27]. In total, thirteen cases of iTTP being successfully treated by caplacizumab have been recently reported in children, with a faster normalization of their platelet counts and favorable outcomes [15,17,18,20,22,23,24]. The pediatric dosing recommendations were developed using model-based simulations and the results of this modeling and simulation analysis constituted the basis for the European extension of indication for caplacizumab (10 mg) to children over 12 years with a body weight of ≥40 kg [29].

Platelet transfusions are relatively contraindicated in children with iTTP and should be limited to the treatment of life-threatening bleeding [13,20].

Thus, several child-onset iTTP cases have reported similar therapeutic experiences when compared to adults over the past 20 years.

### 2.3. Prognosis

Before the caplacizumab era, the mortality rate of the first iTTP episode was ~4% in children [13,21,25]. These epidemiological data need to be updated in the coming years.

Disease relapse is recognized as a risk in iTTP. Child-onset iTTP requires a long-term follow-up to avoid a clinical relapse that is preventable by preemptive rituximab injections when the ADAMTS13 activity drops below 10 IU/dL [7,13,27].

Physical examinations and biological (hemoglobin levels, platelet counts, ADAMTS13 activity monitoring, and autoimmunity) and psychological follow-ups are recommended to evaluate the emergence of autoimmune diseases and the physical and/or psychological sequelae of the disease. Some children have neurologic or renal sequelae. Some of them need psychomotor support and others have familial, social, schooling, or working difficulties. Similar to other autoimmune diseases, iTTP is a life-long disorder with potential psychological, cognitive, and social consequences [13,21].

The pathogenesis of an autoimmune disorder is considered to be multifactorial and a strong association between the HLA region, the generation of autoantibodies against self-antigens, and autoimmune diseases has been described [30]. In children, HLA-DRB1*11 may be a susceptibility factor for iTTP, while HLA-DRB1*04, when not associated with HLA-DQB1*03, may be protective [31]. Pediatricians should also be aware of the occurrence of another systemic autoimmune disease many years following remission [13,32]. Systemic lupus erythematosus is the most common additional autoimmune disorder that has been reported in older girls, in line with the increased frequency of autoimmune disorders at the beginning of puberty.

The transition period from childhood to adulthood is usually difficult and presents many challenges for many young adults with a past history of iTTP. Transition programs are necessary and should include specific actions that patients consider to be priorities, including awareness about relapse prevention, the occurrence of comorbidities (ischemic strokes or other cardiovascular events, hypertension, becoming overweight, etc.), and pregnancy planning.

## 3. Immune Mediated Thrombotic Thrombocytopenic Purpura in Older Patients

The prevalence of older patients with iTTP is increasing due to the aging of the general population and the possibly of improvements in disease recognition. Recent, dedicated studies [33,34,35] have underlined that older iTTP patients have an atypical clinical presentation and a poorer response to treatment and prognosis, which are detailed hereafter.

### 3.1. Clinical Presentation and Diagnosis

As expected, older patients more often present with comorbidities, especially cardiovascular diseases, diabetes, and osteoporosis. The conditions associated with iTTP more frequently involve cancer than autoimmune diseases, while infectious triggers do not seem to be more prevalent [33,34].

Delirium and behavioral abnormalities are often at the forefront of iTTP’s clinical presentation in older patients, as opposed to headache and abdominal pain, which are more frequent in younger patients. Renal and cardiac involvement are more frequent and severe in older patients, whereas hematologic features such as thrombocytopenia and anemia are less pronounced [33], despite a seemingly increased gastro-enteral bleeding rate [35].

These differences translate into poorer performances of both the French and PLASMIC scores [33,36] and a longer time from admission to diagnosis for older patients (3 versus 1 day) [33,35], even though the proportion of iTTP amongst TMA does not seem to decrease with advanced age [35]. Recently, proteinuria and blood pressure were reported as potential leads for improving the performances of these scores [37,38,39,40], but these results warrant confirmation and their added value for older patients more frequently affected by hypertension, diabetes mellitus, and/or chronic kidney disease remains to be evaluated.

### 3.2. Treatment

The current standard of care for iTTP patients relies on TPE, immunosuppressive therapies with corticosteroids and rituximab, and the anti-von Willebrand factor caplacizumab [41]. In the French National Registry, treatment decisions do not differ between older and younger patients, although older patients receive fewer rituximab doses [33] and corticosteroids are less prescribed for patients ≥65 years in the Milan TTP Registry [42].

The combination of TPE and immunosuppressive therapies has resulted in a dramatic improvement in the outcome of iTTP acute episodes [20,41]. Nevertheless, several cohorts with diverse populations have shown that short-term mortality remains significant in older compared to younger iTTP patients (37% versus 9% in one month), due to the higher risks of renal, cardiac, and neurological events [33,43,44], as well as unresponsiveness [45,46]. These studies were published before the caplacizumab era and it would be interesting to evaluate the impact of this new therapeutic approach on the age-related short-term mortality of iTTP patients. In a cluster analysis of 666 patients from the Optum-Humedica database, older patients formed two clusters, with a higher mortality and episodes of a longer duration [47].

A decreased efficacy or increased side effects of caplacizumab have never been described [20,28,41], even though the risk of gastrointestinal or intracranial bleeding calls for caution in frail patients. In a retrospective series of four patients with intracranial hemorrhages after caplacizumab therapy, only one was over 60 years of age [48].

Polypharmacy [34] represents a risk factor for drug interactions and warrants medication optimization upon discharge from the hospital. Particular attention should be paid to antiplatelet agents and anticoagulants, which are more often prescribed to older patients [33], as such treatments could increase the risk of bleeding in association with caplacizumab.

Catheter self-removal as a result of delirium is also an important issue in older iTTP patients; thus, attention should be paid to limit the duration of central venous access to the strict minimum, i.e., it is usually limited to the period of the therapeutic plasma exchange [33].

Current guidelines recommend starting TPE and steroids for patients with TMA after an evaluation of the pretest probability of iTTP, based on a clinical judgement or a risk assessment model [7]. In the elderly, however, poorer diagnostic score performances may render an iTTP diagnosis challenging for clinicians. In older patients with an intermediate probability (French score = 1 or PLASMIC score 5) and/or an atypical clinical presentation, we consider that treatment decisions should rely on knowledge of the older patients’ clinical specificities, a thorough evaluation of alternate diagnoses, and expert opinions. As the proportion of intermediate diagnostic probabilities is increased in elderly patients, clinicians should initiate TPE in these patients even in the presence of an atypical presentation, given the increased risk of short-term mortality and in spite of the increased risk of treatment-related complications.

### 3.3. Prognosis

After an acute episode, relapse rates do not seem to differ between older and younger iTTP patients [33]. However, a growing body of evidence has demonstrated that, in general, iTTP patients require a long-term follow-up due to late-occurring complications. Life expectancy is decreased in iTTP survivors and cardiovascular and neurological complications can occur independently of relapses [49,50,51,52,53]. The pathophysiology of these late complications is thought to rely on both the sequelae of microvascular thrombosis during the acute phase and also a subnormal, non-severe, chronic ADAMTS13 deficiency, with an accumulation of hyper-adhesive, ultra-large VWF multimers released from the endothelium, leading to subclinical vasculopathy and cumulative vascular injury [51]. This last finding appears to be of particular importance in the elderly population and suggests that the risk of cardiac and cerebrovacular events could be mitigated by the careful control of the cardiovascular risk factors and a strict monitoring of the ADAMTS13 activity in remission with the use of preemptive rituximab.

In addition, the psychological, cognitive, and social consequences of iTTP are being increasingly recognized [54,55,56]. Even though there are currently no data comparing older and younger iTTP patients’ cognitive prognoses, older patients are likely to experience a more severe cognitive decline. Delirium, which is more frequent as a presenting feature in older iTTP patients, has been consistently associated with long-term cognitive decline in other contexts [57]. Subclinical vasculopathy related to large, circulating von Willebrand factor multimers may also accelerate this cognitive decline, as evidenced in population cohorts [58]. It is important to note that 26% of patients > 60 years are institutionalized 1 year after their initial episode of iTTP [33].

Infectious complications after the resolution of an iTTP flare-up are scarce, but one worry is that the immunosuppressive effects of corticosteroids and rituximab may lead to a clinically significant infectious risk for older individuals and for those with comorbidities, as shown with other systemic autoimmune diseases [59].

Consistently, long-term survival rates are poorer for older iTTP survivors compared to the general older population (a multivariable HR for death of 3.44, 95% CI [2.02; 5.87]) [33], and these patients experience more frequent long-term cardiovascular and cerebrovascular diseases [34]. Moreover, alike to younger patients, autoimmune diseases can occur months or years after an iTTP diagnosis and warrant a specific follow-up [34,60]. Lastly, older patients have a poorer understanding of the disease, potentially impacting their adherence to the follow-up and their identification of high-risk situations [61]. These data argue for a comprehensive geriatric assessment of older iTTP survivors in order to identify and manage the modifiable risk factors of poor long-term outcomes accordingly (Table 1).

## 4. Conclusions

**Figure 3:**
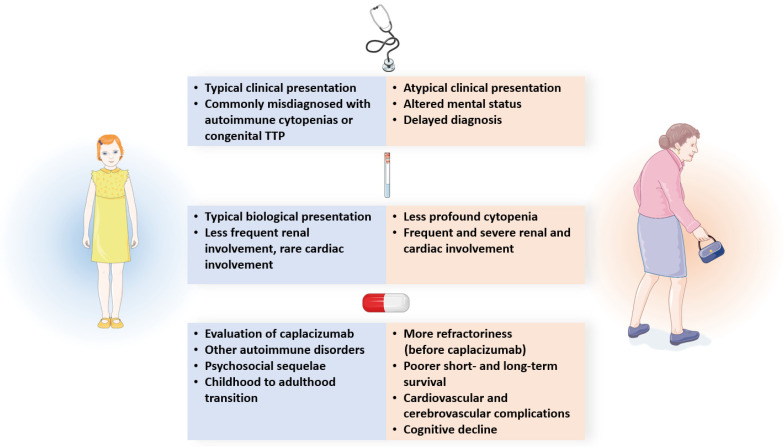
Difference in clinical presentation, biological features, treatment, and outcomes between immune-mediated thrombotic thrombocytopenic purpura at extreme ages.

An iTTP diagnosis can be challenging at extreme ages, resulting in frequently delayed diagnoses. The prediction scores of iTTP are not used for children and have a lower sensitivity and specificity in patients over 60 years old. ADAMTS13 therefore remains the unique biological marker that is able to definitely confirm the diagnosis of iTTP in these patients. The treatment of iTTP in children and elderly patients should not differ from that in other age groups. In both populations, an assessment of caplacizumab’s efficacy and tolerance is urgently needed. While the efficacy and safety of caplacizumab in children are likely to be similar to those of the drug in adults, additional safety data are required for the elderly, where patients are typically polymedicated and more exposed to bleeding complications. In all cases, long-term follow-up is crucial to preventing relapses of the disease, to identifying the occurrence of systemic autoimmune disorders, and to evaluating its consequences for social life. In children, helping patients during their transition period to adulthood is key (Figure 3).

## Figures and Tables

**Table 1:** Recommendations and research agenda (italic) for optimal care of iTTP in older patients.

	Recommendations
Diagnosis	Physician awareness of atypical neurological presentations
	Adaptation of clinical scores for older patients specificities
Treatment	Rapid identification and intensification in unresponsive patients Medication optimization to avoid drug interactions Reassessment of prognostic factors in the caplacizumab era
Long-term follow-up	Comprehensive geriatric assessment and management of associated (cardiovascular) risk factors Careful ADAMTS13 monitoring ± preemptive rituximab
	Characterization of cognitive impairment in older patients following iTTP episode

## Data Availability

Not applicable.
